# Deletion of the GluA1 AMPA receptor subunit alters the expression of short-term memory

**DOI:** 10.1101/lm.2014911

**Published:** 2011-03

**Authors:** David J. Sanderson, Rolf Sprengel, Peter H. Seeburg, David M. Bannerman

**Affiliations:** 1 Department of Experimental Psychology, University of Oxford, Oxford OX1 3UD, United Kingdom; 2 Department of Molecular Neurobiology, Max-Planck Institute of Medical Research, D-69120 Heidelberg, Germany

## Abstract

Deletion of the GluA1 AMPA receptor subunit selectively impairs short-term memory for spatial locations. We further investigated this deficit by examining memory for discrete nonspatial visual stimuli in an operant chamber. Unconditioned suppression of magazine responding to visual stimuli was measured in wild-type and GluA1 knockout mice. Wild-type mice showed less suppression to a stimulus that had been presented recently than to a stimulus that had not. GluA1 knockout mice, however, showed greater suppression to a recent stimulus than to a nonrecent stimulus. Thus, GluA1 is not necessary for encoding, but affects the way that short-term memory is expressed.

The GluA1 subunit of the AMPA receptor is a key mediator of hippocampal synaptic plasticity (Zamanillo et al. 1999). It is especially important for a short-lasting, rapidly induced form of synaptic plasticity (Hoffman et al. 2002; Romberg et al. 2009; Erickson et al. 2010). Moreover, there is converging evidence that GluA1 is necessary for short-term memory but not for long-term memory. For example, mice lacking GluA1 (GluA1^−/−^ mice) form long-term associations between spatial locations and rewards (such as food or escape from water), but fail to discriminate spatial locations on the basis of how recently they have been experienced in spatial win–shift tasks (Reisel et al. 2002; Schmitt et al. 2003; see Sanderson and Bannerman 2011b). Similarly, GluA1^−/−^ mice show long-term spatial recognition, but impaired short-term recognition memory (Sanderson et al. 2007, 2009).

To further investigate the role of GluA1 in short-term memory we tested whether the GluA1 knockout deficit extended to discrete nonspatial visual stimuli in a spontaneous recognition memory task. Typically, rodents show behaviors such as orienting to novel visual stimuli, but these unconditioned behaviors habituate over time as the visual stimuli become increasingly familiar (e.g., Hall and Channell 1985; Jordan et al. 2000). We used suppression of nose-poke responding as an indirect measure of unconditioned responding to the visual stimuli (e.g., Robinson et al. 2009). Thus, mice were allowed to collect food rewards from a magazine in an operant chamber and the reduction in magazine activity (i.e., nose poking) when visual stimuli were presented was measured. To specifically examine the role of short-term memory mice received trials that consisted of a series of two stimulus presentations. Within a trial one stimulus was presented, followed 30 sec later by a second stimulus. The second stimulus was either the same as the first (condition Same), or different from the first (condition Different). By using this manipulation it is possible to test stimulus-specific effects of short-term memory on responding to the visual stimuli. For example, if short-term memory reduces the unconditioned response to a stimulus then mice should show less suppression when the second stimulus of a trial is the same as the first, compared with when the first and second stimuli are different.

The experiment used littermate, age-matched wild-type (female, *N* = 4; male, *N* = 4) and GluA1^−/−^ mice (female, *N* = 4; male, *N* = 4), bred in the Department of Experimental Psychology, University of Oxford (for details of genetic construction, breeding, and subsequent genotyping, see Zamanillo et al. [1999]). Mice were caged in same-sex groups of two to seven, in a temperature-controlled housing room on a 12-h light/dark cycle (0700–1900), and had ad libitum access to water. Mice were approximately 6 mo old at the start of testing. Mice were maintained at 85% of their free-feeding weight throughout testing by receiving a restricted diet.

Mice were tested in operant chambers (15.9 × 14.0 × 12.7 cm; ENV-307A, Med Associates), enclosed in sound-attenuating cubicles (ENV-022MD, Med Associates) using Med-PC IV software (Med Associates). Sucrose pellets (20 mg; TestDiet, ETH) were dispensed into a magazine (2.9 × 2.5 × 1.9 cm; ENV-303M, Med Associates). Breaks in an infrared beam (ENV-303HDM, Med Associates) across the bottom of the magazine were used to measure magazine activity. The two visual stimuli used were (stimulus A) illumination of the house light (ENV-315M, Med Associates) that was located on the wall opposite the magazine; and (stimulus B) illumination of two flashing (1 sec on, 1 sec off) LEDs (ENV-321M, Med Associates) that were located at an equal distance to the left and right above the magazine. Each stimulus was 10 sec in duration. Each chamber was equipped with a fan (ENV-025AC, Med Associates) that was turned on for the duration of the session.

Prior to testing, mice received five sessions of magazine training. In the first session pellets were dispensed on a variable time schedule (VT) of 60 sec for 1 h. On the four subsequent sessions pellets were dispensed on a VT-120 sec for 30 min. The mean level of responding during the final session was 7.2 sec of magazine activity per min.

During testing 15 single pellets were dispensed on a VT-120 sec (range 30–210 sec) each session. The delivery of pellets was uncorrelated with magazine activity and visual stimuli. Mice received trials in which pairs of visual stimuli were presented. One stimulus was initially presented and then a second stimulus was presented 30 sec later. Each session consisted of four trials. Within a session, for half of the trials the pair of stimuli were the same (condition Same, i.e., A followed by A, or B followed by B) and for the other half of the trials the pair of stimuli were different (condition Different, i.e., A followed by B, or B followed by A). Also, for half of the trials of each condition the first stimulus was A, and for the other half of the trials the first stimulus was B. The intertrial interval was 310 sec. The first trial of each session commenced after 310 sec. The trial order was pseudorandom across sessions, and within each session the trial order was counterbalanced across genotype and sex. The cumulative time that mice spent with their heads in the magazine was recorded for each stimulus presentation (first stimulus and second stimulus), and for the equivalent period of time prior to stimulus presentations (pre-first stimulus, pre-second stimulus). For each epoch the cumulative duration of magazine activity is expressed as a percentage of 10 sec.

The mean percentage of magazine activity during the pre-first stimulus and pre-second stimulus periods are shown in Table 1. Data are blocked by four sessions to compare performance during the first (block 1) and second (block 2) halves of training. An analysis of variance (ANOVA) that included genotype, prestimulus period, and block as factors failed to reveal any significant main effects (*F*-values <1) or interactions (*P*-values >0.1). Thus, wild-type and GluA1^−/−^ mice showed similar levels of baseline magazine activity that were stable across training. For each mouse the mean of the pre-first stimulus and pre-second stimulus periods in each block was used as a baseline measure with which to compare magazine activity during stimulus presentations.

**Table 1.** Mean magazine activity (±SEM) for prestimulus periods for block 1 (sessions 1–4) and block 2 (sessions 5–8)

	Block 1		Block 2	
	Pre-first stimulus	Pre-second stimulus	Pre-first stimulus	Pre-second stimulus
WT	5.6 ± 1.3	5.0 ± 0.5	9.6 ± 2.8	9.1 ± 1.5
GluA1^−/−^	8.0 ± 2.8	8.4 ± 2.8	6.8 ± 2.6	7.1 ± 2.7

Magazine activity is shown as a percentage of 10 sec.

To simplify the analysis of responding during the stimulus presentations magazine activity was converted to a difference score by subtracting the percentage of magazine activity during the stimulus from the percentage of magazine activity during the prestimulus, baseline period. Thus, scores greater than zero indicated that the stimulus presentation resulted in suppression of responding, whereas scores equal to zero indicated no change in responding. The difference scores for the first stimulus of a trial (collapsed across conditions Same and Different) and the second stimuli in condition Same and condition Different, for blocks 1 and 2, are shown in Figure 1. The left half of Figure 1 shows the results for wild-type mice. Whereas the first stimulus elicited a high level of suppression, this effect was reduced when the second stimulus was the same as the first, but not when it was different from the first. This indicates that a recent presentation of a stimulus resulted in stimulus-specific habituation of suppression. The results for GluA1^−/−^ mice are shown on the right half of Figure 1. The GluA1^−/−^ mice showed a markedly different pattern of performance compared with wild-type mice. Suppression to the first stimulus was somewhat weaker for GluA1^−/−^ mice than for wild-type mice. However, suppression was enhanced if the second stimulus was the same as the first, but this was not true if the second stimulus was different from the first. This indicates that a recent stimulus presentation resulted in stimulus-specific sensitization of suppression. For both groups overall levels of suppression were weaker in block 2 than in block 1 indicating long-term habituation of suppression.

**Figure 1.**
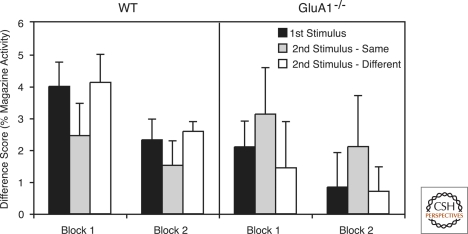
Mean difference scores (prestimulus magazine activity − stimulus magazine activity) for the first stimulus of a trial (collapsed across conditions Same and Different), and the second stimulus of a trial in condition Same and condition Different across blocks 1 and 2. Positive scores indicate suppression of responding and a score of zero indicates no change in responding. Magazine activity is shown as a percentage of 10 sec. Error bars indicate SEM. Results for wild-type mice (WT) are shown on the *left*. A recent stimulus exposure resulted in habituation of suppression when the second stimulus was the same as the first, but not when the stimuli were different. Results for GluA1^−/−^ mice are shown on the *right*. A recent stimulus exposure resulted in enhanced suppression when the second stimulus was the same as the first, but not when the stimuli were different.

A 2 (genotype) × 2 (block) × 3 (stimulus: first stimulus, second stimulus – same, second stimulus – different) ANOVA confirmed that there was a significant effect of block (*F*_(1,14)_ = 4.69, *P* < 0.05), and importantly, a significant stimulus by genotype interaction (*F*_(2,28)_ = 9.68, *P* < 0.002; all other main effects and interactions were not significant, *F*-values <1). To explore the nature of this interaction two separate analyses were performed to answer specific questions. First, to examine whether repetitions of the same stimulus within a trial led to a reduction of suppression, the difference scores for the first stimulus and the second stimulus – same were compared. It was found that there was a significant stimulus by genotype interaction (*F*_(1,14)_ = 9.91, *P* < 0.01). Simple main effects analysis of the interaction using the error term from the original ANOVA confirmed that for wild-type mice suppression was significantly weaker on the second stimulus presentation than on the first (*F*_(1,14)_ = 5.05, *P* < 0.05), but for GluA1^−/−^ mice suppression was significantly greater on the second stimulus presentation than on the first (*F*_(1,14)_ = 4.86, *P* < 0.05). There was no significant effect of genotype for first or second stimuli (*P*-values >0.1).

To assess whether the observed habituation and sensitization effects were stimulus-specific the difference scores for the second stimuli in condition Same and condition Different were compared. Once again there was a significant stimulus by genotype interaction (*F*_(1,14)_ = 12.84, *P* < 0.005). Simple main effects analysis of the interaction demonstrated that while suppression was significantly weaker in condition Same than in condition Different for wild-type mice (*F*_(1,14)_ = 5.671, *P* < 0.04), it was significantly greater in condition Same than in condition Different for GluA1^−/−^ mice (*F*_(1,14)_ = 7.22, *P* < 0.02). This pattern of results is clearly illustrated by subtracting the percentage of magazine activity for condition Different from the percentage of magazine activity for condition Same (see Fig. 2). Thus, positive difference scores indicate less suppression in the condition Same than in the condition Different (i.e., habituation) and negative scores indicate the opposite effect (i.e., sensitization). Whereas a recent stimulus exposure resulted in stimulus-specific habituation in wild-type mice, it resulted in stimulus-specific sensitization in GluA1^−/−^ mice.

**Figure 2.**
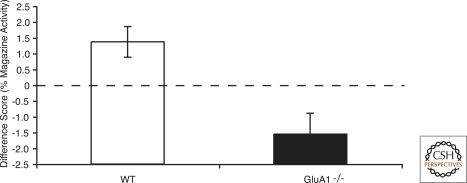
Magazine activity in condition Same and condition Different shown as a difference score (Same − Different). Positive scores indicate stimulus-specific habituation of suppression, and negative scores indicate stimulus-specific sensitization of suppression. The dashed line indicates chance performance. Magazine activity is shown as a percentage of 10 sec. Error bars indicate ± SEM.

These results demonstrate that GluA1 is important for short-term visual memory. This finding adds to previous research demonstrating impaired short-term memory for spatial locations (Sanderson et al. 2007, 2009) in GluA1^−/−^ mice. Thus, GluA1 plays a role in short-term memory that is general to stimuli from different domains. Importantly, the results provide new insight into the nature of the short-term memory deficit in GluA1^−/−^ mice. Both groups showed an ability to discriminate between stimuli on the basis of how recently they had been presented, but they expressed this information differently. This demonstrates that GluA1 is not necessary for discriminating between the visual cues. Furthermore, the pattern of results with GluA1^−/−^ mice does not occur because they fail to encode or store the short-term memory.

GluA1^−/−^ mice discriminate between recent and nonrecent stimuli in a qualitatively different manner to control mice. This indicates that GluA1 is necessary for the expression of short-term memory. In wild-type mice a short-term memory for a stimulus weakened the unconditioned response and thus reduced suppression. However, in GluA1^−/−^ mice short-term memory had the opposite effect: The unconditioned response was stronger for a recent stimulus than for a nonrecent stimulus, resulting in greater suppression in condition Same than in condition Different. This suggests that short-term memory can have different effects on unconditioned responding to stimuli and that GluA1 plays an important role in the expression of short-term memory.

An account of memory proposed by Wagner (1981) provides a potential explanation for the opposite effects of short-term memory in wild-type and GluA1^−/−^ mice. Wagner suggested that when a stimulus is presented a representation of the stimulus increasingly enters a primary activity state before decaying into a secondary activity state where it remains before eventually returning to an inactive state. Whereas a stimulus representation can elicit strong levels of responding in the primary activity state, the level of responding is weak in the secondary activity state. Although a representation will move from the primary to the secondary state with the passage of time, stimulus representations in the secondary state cannot return directly to the primary state. Therefore, if a stimulus is presented while its representation is in the secondary state then the stimulus will be less able to evoke responding (i.e., habituation will occur). However, this will not be the case for a stimulus whose representation is not in the secondary state. This description of memory provides an account of the stimulus-specific, short-term habituation effect that was seen in control mice in the present study.

For GluA1^−/−^ mice short-term memory had a positive, rather than a negative effect on unconditioned responding. Based on Wagner's explanation of the habituation effect in controls, a possible account of the performance of GluA1^−/−^ mice is that a recent stimulus presentation increased activity in the primary activity state. Thus, for GluA1^−/−^ mice the stimulus representation may not have transferred efficiently to the secondary state. Subsequently, when the same stimulus is presented there is an increase in responding.

Consistent with this we have argued previously that GluA1 deletion reduces the rate at which stimulus representations transfer from the primary to the secondary activity state (Sanderson et al. 2009). This hypothesis is supported by the impaired performance of GluA1^−/−^ mice on short-term recognition for recent stimuli (Sanderson et al. 2007, 2009). Moreover, this account makes the prediction that GluA1 deletion, while impairing short-term memory, will leave associative learning intact. This is because associations are formed between stimulus representations that are coactive in the primary state (Wagner 1981). Indeed, there are numerous examples of preserved associative learning in GluA1^−/−^ mice (Zamanillo et al. 1999; Reisel et al. 2002; Schmitt et al. 2003).

Wagner's model also makes the prediction that short-term and long-term memory will compete under certain circumstances (e.g., Sanderson and Bannerman 2011a). For example, if the interval between presentations of a conditioned stimulus (CS) is short, then less conditioning may occur in wild-type animals because by the time of the second CS presentation, the representation will be in the secondary state. This will reduce the amount of associative learning that can occur (see Best and Gemberling 1977; Sunsay et al. 2004). Thus, it follows that if GluA1 deletion decreases the rate at which representations enter the secondary state then, under certain conditions, GluA1 deletion should enhance long-term memory. We have recently provided evidence to support this prediction. GluA1^−/−^ mice show enhanced long-term spatial memory despite impaired short-term spatial memory (Sanderson et al. 2009; for review, see Sanderson et al. 2010).

It is worth noting that the present results are similar to those found with hippocampal lesions in rats (Marshall et al. 2004). In the study by Marshall et al., rats received simultaneous presentation of two different lights (constant light, flashing light). One of the lights had been presented more recently than the other. It was found that control rats were more likely to orient to the less recently presented light than the more recently presented light. However, hippocampal-lesioned rats showed the opposite effect; exhibiting greater orienting toward the more recently presented light than the less recently presented light. This result is important in that it demonstrates that the memory representations for visual stimuli are not stored in the hippocampus (see also Honey et al. 1998; Honey and Good 2000a,b), but suggests a role for the hippocampus in expression of memory (Gray and McNaughton 2000). The present data show that short-term memory expression is GluA1-dependent as well as hippocampus-dependent. Collectively, the results of Marshall et al. (2004) and the present results provide a dissociation of memory expression that is uniquely accounted for by Wagner's (1981) model of short-term memory.

In conclusion, the present results provide a novel demonstration that GluA1 deletion impairs short-term visual memory. Strikingly, this deficit occurs because short-term memory has a qualitatively different impact on behavior in GluA1^−/−^ mice than in control mice. Thus, GluA1 is not necessary for encoding or storage, but is necessary for the expression of short-term memory.

## References

[SANDERSONLM20149C1] Best MR, Gemberling GA 1977 Role of short-term processes in the conditioned stimulus preexposure effect and the delay of reinforcement gradient in long-delay tast-aversion learning. J Exp Psychol Anim Behav Process 14: 219–234

[SANDERSONLM20149C2] Erickson MA, Maramara LA, Lisman J 2010 A single brief burst induces GluR1-dependent associative short-term potentiation: A potential mechanism for short-term memory. J Cogn Neurosci 22: 2530–2540 19925206 10.1162/jocn.2009.21375 PMC3195522

[SANDERSONLM20149C3] Gray JA, McNaughton N 2000 The neuropsychology of anxiety. Oxford University Press, Oxford, UK

[SANDERSONLM20149C4] Hall G, Channell S 1985 Differential effects of contextual change on latent inhibition and on the habituation of an orienting response. J Exp Psychol Anim Behav Process 11: 470–481

[SANDERSONLM20149C5] Hoffman DA, Sprengel R, Sakmann B 2002 Molecular dissection of hippocampal theta-burst pairing potentiation. Proc Natl Acad Sci 99: 7740–7745 12032353 10.1073/pnas.092157999 PMC124338

[SANDERSONLM20149C6] Honey RC, Good M 2000a Associative components of recognition memory. Curr Opin Neurobiol 10: 200–204 10753791 10.1016/s0959-4388(00)00069-6

[SANDERSONLM20149C7] Honey RC, Good M 2000b Associative modulation of the orienting response: Distinct effects revealed by hippocampal lesions. J Exp Psychol Anim Behav Process 26: 3–14 10650540 10.1037//0097-7403.26.1.3

[SANDERSONLM20149C8] Honey RC, Watt A, Good M 1998 Hippocampal lesions disrupt an associative mismatch process. J Neurosci 18: 2226–2230 9482806 10.1523/JNEUROSCI.18-06-02226.1998 PMC6792925

[SANDERSONLM20149C9] Jordan WP, Strasser HC, McHale L 2000 Contextual control of long-term habituation in rats. J Exp Psychol Anim Behav Process 26: 323–339 10913996 10.1037//0097-7403.26.3.323

[SANDERSONLM20149C10] Marshall VJ, McGregor A, Good M, Honey RC 2004 Hippocampal lesions modulate both associative and nonassociative priming. Behav Neurosci 118: 377–382 15113263 10.1037/0735-7044.118.2.377

[SANDERSONLM20149C11] Reisel D, Bannerman DM, Schmitt WB, Deacon RM, Flint J, Borchardt T, Seeburg PH, Rawlins JN 2002 Spatial memory dissociations in mice lacking GluR1. Nat Neurosci 5: 868–873 12195431 10.1038/nn910

[SANDERSONLM20149C12] Robinson J, Sanderson DJ, Aggleton JP, Jenkins TA 2009 Suppression to visual, auditory, and gustatory stimuli habituates normally in rats with excitotoxic lesions of the perirhinal cortex. Behav Neurosci 123: 1238–1250 20001107 10.1037/a0017444 PMC4231296

[SANDERSONLM20149C13] Romberg C, Raffel J, Martin L, Sprengel R, Seeburg PH, Rawlins JN, Bannerman DM, Paulsen O 2009 Induction and expression of GluA1 (GluR-A)-independent LTP in the hippocampus. Eur J Neurosci 29: 1141–1152 19302150 10.1111/j.1460-9568.2009.06677.x PMC2695863

[SANDERSONLM20149C14] Sanderson DJ, Bannerman DM 2011a Competitive short-term and long-term memory processes in spatial habituation. J Exp Psychol Anim Behav Process (in press) 10.1037/a0021461 PMC3085505 21319917

[SANDERSONLM20149C15] Sanderson DJ, Bannerman DM 2011b The role of habituation in hippocampus-dependent spatial working memory tasks: Evidence from GluA1 AMPA receptor subunit knockout mice. Hippocampus (in press). doi: 10.1002/hipo.20896 10.1002/hipo.20896 PMC3490380 21125585

[SANDERSONLM20149C16] Sanderson DJ, Gray A, Simon A, Taylor AM, Deacon RM, Seeburg PH, Sprengel R, Good MA, Rawlins JN, Bannerman DM 2007 Deletion of glutamate receptor-A (GluR-A) AMPA receptor subunits impairs one-trial spatial memory. Behav Neurosci 121: 559–569 17592947 10.1037/0735-7044.121.3.559

[SANDERSONLM20149C17] Sanderson DJ, Good MA, Skelton K, Sprengel R, Seeburg PH, Rawlins JN, Bannerman DM 2009 Enhanced long-term and impaired short-term spatial memory in GluA1 AMPA receptor subunit knockout mice: Evidence for a dual-process memory model. Learn Mem 16: 379–386 19470654 10.1101/lm.1339109 PMC2704103

[SANDERSONLM20149C18] Sanderson DJ, McHugh SB, Good MA, Sprengel R, Seeburg PH, Rawlins JN, Bannerman DM 2010 Spatial working memory deficits in GluA1 AMPA receptor subunit knockout mice reflect impaired short-term habituation: Evidence for Wagner's dual-process memory model. Neuropsychologia 48: 2303–2315 20350557 10.1016/j.neuropsychologia.2010.03.018 PMC2938569

[SANDERSONLM20149C19] Schmitt WB, Deacon RM, Seeburg PH, Rawlins JN, Bannerman DM 2003 A within-subjects, within-task demonstration of intact spatial reference memory and impaired spatial working memory in glutamate receptor-A-deficient mice. J Neurosci 23: 3953–3959 12736365 10.1523/JNEUROSCI.23-09-03953.2003 PMC6742186

[SANDERSONLM20149C20] Sunsay C, Stetson L, Bouton ME 2004 Memory priming and trial spacing effects in Pavlovian learning. Learn Behav 32: 220–229 15281394 10.3758/bf03196023

[SANDERSONLM20149C21] Wagner AR 1981 SOP: A model of automatic memory processing in animal behavior. In Information processing in animals: Memory mechanisms (ed. Spear NE, Miller RR), pp. 5–47 Lawrence Erlbaum Associates Inc, Hillsdale, NJ

[SANDERSONLM20149C22] Zamanillo D, Sprengel R, Hvalby O, Jensen V, Burnashev N, Rozov A, Kaiser KM, Koster HJ, Borchardt T, Worley P, 1999 Importance of AMPA receptors for hippocampal synaptic plasticity but not for spatial learning. Science 284: 1805–1811 10364547 10.1126/science.284.5421.1805

